# Perceptions of psychiatrists toward long-acting injectable antipsychotics: A cross-sectional survey from Fujian, China

**DOI:** 10.1371/journal.pone.0362055

**Published:** 2026-11-02

**Authors:** Yixiang Zhou, Weijun Gu, Zhiqiang Cui, Yinghua Huang

**Affiliations:** 1 Department of Pharmacy, Xiamen Xianyue Hospital, Xiamen, China; 2 Xianyue Hospital Affiliated with Xiamen Medical College, Xiamen, China; 3 Fujian Psychiatric Center, Xiamen, China; 4 Fujian Clinical Research Center for Mental Disorders, Xiamen, China; 5 Department of Psychiatry, Xiamen Xianyue Hospital, Xiamen, China; NYU Grossman School of Medicine: New York University School of Medicine, UNITED STATES OF AMERICA

## Abstract

**Objective:**

This study aimed to assess the knowledge, attitudes, and willingness of psychiatrists to promote long-acting injectable antipsychotics (LAIs) in Fujian Province, China, and to identify the influencing factors.

**Methods:**

A cross-sectional survey was conducted using convenience sampling among psychiatrists attending a provincial conference. An 18-item online questionnaire was distributed via *Wenjuanxing*. Descriptive statistics, Mann-Whitney U tests and Spearman’s rank correlation were used. Benjamini-Hochberg FDR correction was applied for multiple correlations

**Results:**

A total of 222 psychiatrists completed the survey, with 90 respondents (40.5%) identifying as female. The largest age group was 30–39 years (46.9%). Most participants (51.4%) had > 10 years of clinical experience, and 65 (29.3%) held senior professional titles. Most participants reported adequate knowledge of LAIs (96.4%), recognized their therapeutic value (80.2%) and favorable application prospects (87.8%), and expressed a willingness to promote LAIs (82.4%).High cost was identified as the primary barrier to clinical use (90.5%) and the most critical factor for wider adoption (88.3%). Psychiatrists with more clinical experience (>10 years) held significantly more positive attitudes regarding overall therapeutic value, willingness to promote LAIs, and support for early application (all *P* < 0.05). Spearman’s correlation analysis indicated that positive attitudes were significantly associated with greater clinical experience (ρ = 0.302, *q* < 0.01) and higher levels of knowledge (ρ = 0.297, *q* < 0.01) after FDR correction.

**Conclusion:**

Psychiatrists in Fujian Province demonstrated adequate knowledge and generally positive attitudes toward LAIs. Psychiatrists’ willingness to promote LAIs was associated with knowledge level and clinical experience, but reducing the cost of drug patents may contribute to improving the prescription rates. Future efforts should focus on systematic training, academic communication, positive prescribing experiences, and policy coordination to overcome cost and stigma barriers.

## 1. Introduction

Schizophrenia is a chronic, relapsing, and severely disabling mental disorder that affects approximately 1% of the global population, and imposes a substantial socioeconomic burden on patients, families, and society [1]. In China, the lifetime prevalence of schizophrenia is reported to be 0.6% [2]. By 2020, the national registry documented 6.43 million individuals with severe mental disorders, of whom approximately 4.5 million were diagnosed with schizophrenia [3]. However, these figures may be underestimated because of regional disparities in healthcare resources and accessibility [4]. A key therapeutic challenge is antipsychotic nonadherence, which is attributable to a lack of insight into illness, premature discontinuation following symptom improvement, cognitive impairment, and adverse effects [5].

Inadequate adherence increases the risk of relapse nearly fivefold compared with that associated with consistent adherence [6,7]. Furthermore, 40%−60% of patients experience at least one relapse, which is associated with heightened rehospitalization rates, functional deterioration, increased suicide risk, and increased healthcare costs [8,9]. These outcomes underscore the importance of improving medication adherence and ensuring continuous, comprehensive care for individuals living with schizophrenia.

Long-acting injectable antipsychotics(LAIs), particularly second-generation antipsychotic LAIs (SGA-LAIs), increase adherence by reducing dosing frequency and minimizing missed doses and unsupervised discontinuation. SGA-LAIs are associated with improved adherence, reduced relapse and rehospitalization rates, and decreased caregiver burden [10]. Compared with first-generation antipsychotic LAIs, SGA-LAIs offer several advantages, including longer dosing intervals (e.g., monthly administration), more stable plasma concentrations and often no requirement for concomitant oral therapy during initiation [11]. Consequently, international clinical guidelines recommend SGA-LAIs across various treatment stages, particularly for patients with poor adherence or high relapse risk [12,13]. In China, the National Health Commission advocates for the use of LAIs in patients who demonstrate poor treatment adherence, inadequate family supervision, or potential safety risks [14]. Furthermore, LAIs have been included in both the National Essential Medicines List and the National Reimbursement Drug List [15,16]. Some local governments have implemented free LAI provision policies, which have contributed to an 18.6% increase in LAI prescriptions and a 52% reduction in rehospitalization [17].

Despite the well-documented benefits, the prescription rates of LAIs remain lower than expected globally, with considerable regional variation [18–20]. In China, a recent survey reported LAIs prescription rates of only 5.2%, substantially lower than those reported in Japan (16.2%), Spain (51.1%), and France (42.6%) [21]. Factors influencing LAIs utilization included the attitudes of psychiatrists, caregivers, and patients, as well as drug cost and accessibility [22]. Studies have revealed considerable variation among mental health professionals. For instance, a study conducted by Oguchi Yet al [23]. revealed that more than over 50% of psychiatrists in Japan held negative views toward SGA-LAIs. Their concerns included the high cost of the medications, the discomfort associated with injections, the complexity involved in transitioning patients from oral to injectable formulations, and doubts about prior oral adherence. Similarly, a study from India reported that while 54.3% of participants believed that patients had reasonable acceptance of LAIs, 59% acknowledged that LAIs were underutilized [24]. The primary reasons cited were cost (55.5%), patient reluctance (42.9%), irregular drug supply (41.3%), and fear of injections (41.2%). In contrast, a survey of community psychiatrists in Beijing indicated that 80% were willing to recommend LAIs to their patients [25]. Although China’s volume-based procurement policy, introduced in 2019, has significantly reduced medication prices [26], the relatively high cost of SGA-LAIs continues to limit prescription [27].

Given the complex interplay of these factors, there is an urgent need to explore psychiatrists’ perspectives on LAIs to better understand the clinical factors influencing LAIs adoption in a real-world clinical setting. This study aims to (1) quantitatively assess psychiatrists’ overall attitudes toward LAIs, and (2) identify key factors influencing their willingness to prescribe LAIs, including perceptions of benefits, drawbacks, and concerns. The findings are expected to clarify implementation barriers in China, inform targeted educational programs, enhance doctor-patient communication, and guide health policy. Ultimately, this may promote rational LAIs use and improve patients’ quality of life.

## 2. Methods

### 2.1. Study design and participants

The survey questionnaire was developed by the research team following a comprehensive literature review and consideration of local factors influencing prescribing behaviors, including medication costs and healthcare policies. To ensure scientific validity and relevance, the questionnaire was reviewed by an expert panel comprising two senior professors in psychiatry and one biostatistician. The research team refined the questionnaire based on expert feedback. Additionally, a pilot test was conducted with a small sample of participants to assess clarity, comprehensiveness, and feasibility. Initially, 22 items were drafted. An additional 15 psychiatrists (10 from tertiary psychiatric hospitals and 5 from community mental health centers) were invited to participate in a pilot test of the questionnaire. Their characteristics were: mean age 38.5 years, mean years of practice 9.2 years, and 60% were attending physicians. The research team revised and refined the questionnaire based on the suggestions from the experts and pilot participants, finalizing the formal version. Finally, 18 items were retained, and the time required to complete the questionnaire was estimated. The sample size was determined to be 5–10 times the number of variables, and considering a 20% invalid response rate, 113–225 participants were targeted.

The reliability analysis results of the questionnaire were expressed using Cronbach’s αcoefficient. The Cronbach’s αcoefficient for the attitude toward the long-acting injections part of the questionnaire was 0.850, indicating good reliability. The final questionnaire comprised three sections and required approximately 8–10 minutes to complete. The first section, titled “Basic Information,” collected data on participants’ sociodemographic characteristics, including gender, age, number of years in clinical practice, and professional title(e.g., Professor, Associate Professor). The second section focused on knowledge level and clinical practice regarding LAIs. The third section addressed the willingness to promote LAIs and the influencing factors.

### 2.2. Data collection and ethical approval

Data were collected via an online survey administered through Wenjuanxing, a widely used survey platform integrated into WeChat, a popular social media application in China with more than one billion users. The study employed a cross-sectional design with convenience sampling. Participants were recruited from psychiatrists attending the 2025 Fujian Provincial Psychiatry Alliance Summit, which included a substantial proportion of psychiatrists practicing in Fujian Province. Participants received invitations via WeChat by scanning a QR code. All the respondents provided electronic informed consent and participated voluntarily. To protect confidentiality, no personally identifiable information (e.g., names, identification numbers, contact details) was collected. Nonrespondents were not contacted for follow-up. All collected data were handled to maintain confidentiality, and no financial incentives were offered for participation. The study protocol was approved by the Medical Ethics Committee of Xiamen Xianyue Hospital (No.2025-KY-058).

### 2.3. Statistical analysis

Data were collected via Wenjuanxing, exported to Microsoft Excel for cleaning, and analyzed using IBM SPSS Statistics version 26.0 (IBM Corp., Armonk, NY, USA). Descriptive statistics were used to summarize sociodemographic characteristics. Normality was assessed using the Shapiro-Wilk test, which indicated that the data were not normally distributed. Accordingly, non-parametric methods were used: the Mann-Whitney U test for two-group comparisons and Spearman’s rank correlation for associations. Categorical variables are presented as frequencies and percentages. For multiple-choice items, response frequencies and percentages were calculated relative to the total number of participants. To investigate patterns and determine predictors of psychiatrists’ attitudes toward LAIs, the following analytical approaches were used: (1) Subgroup analysis: Participants were dichotomized by clinical experience (≤10 vs. > 10 years), professional title (senior vs. junior), self-assessed LAIs knowledge (sufficient vs. insufficient), and LAIs prescribing experience (experienced vs. limited). Differences in ordinal attitude measures were evaluated using the Mann-Whitney U test, with effect sizes expressed as r values. (2) Correlational analysis: Spearman’s rank correlation was used to examine associations between psychiatrist characteristics (years of practice, professional title, knowledge, and clinical experience) and attitude-related outcomes. The results are reported as Spearman’s ρ coefficients. For all tests, a two-tailed *P* < 0.05 was considered to indicate statistical significance. Given the multiple correlation analyses performed, the Benjamini-Hochberg false discovery rate (FDR) procedure was applied to control for multiple comparisons. The adjusted *q*-values were calculated using the Benjamini-Hochberg method, and a two-tailed *q* < 0.05 was considered statistically significant after FDR correction.

## 3. Results

### 3.1. Demographic characteristics

Among the 222 participating psychiatrists, 90(40.5%) were female. The largest age group was 30–39 years (n = 104, 46.9%). Overall, 114(51.4%) participants had > 10 years of clinical experience, and 65(29.3%) held senior professional titles. The demographic characteristics are shown in Table 1.

**Table 1:** Demographic characteristics of participants (n = 222).

Characteristic	Frequency, *n*(%)
Gender	
Male	132 (59.5)
Female	90 (40.5)
Age	
≤29 years	34 (15.3)
30-39 years	104 (46.9)
40-49 years	52 (23.4)
50-59 years	30 (13.5)
≥60 years	2 (0.9)
Years in practice	
≤5 years	51 (23.0)
6-10 years	57 (25.9)
11-20 years	62 (27.9)
>20 years	52 (23.4)
Professional Title	
Professor	12 (5.4)
Associate Professor	53 (23.9)
Attending Physician	86 (38.7)
Resident (Physician)	71 (32.0)
Self-assessed knowledge of LAIs	
Very knowledgeable	70 (31.5)
Somewhat knowledgeable	144 (64.9)
Limited knowledge	7 (3.2)
Minimal knowledge	1 (0.5)
LAIs prescribing experience	
Frequent prescriber	27 (12.2)
Occasional prescriber	119 (53.6)
Rare prescriber	55 (24.8)
Never prescribed	21 (9.5)

### 3.2. Knowledge level, practice patterns, and overall attitudes

A majority (96.4%) of the participants demonstrated high levels of familiarity with the pharmacological properties and clinical benefits of LAIs, with nearly one-third (31.5%) of the participants rating themselves as “very knowledgeable.” The advantages of improving medication adherence (93.2%) and reducing dosing frequency to enhance patient convenience (94.6%) were widely acknowledged. With respect to perceived limitations, high cost (90.5%) was identified as the primary barrier, followed by low patient acceptance of injections (60.4%).

Most psychiatrists (90.5%) had experience prescribing LAIs. Overall, 65.7% of psychiatrists had some prescribing experience with LAIs (12.2% frequent prescribers and 53.6% occasional prescribers), while 24.8% were rare prescribers and 9.5% never prescribed. The predominant indication for initiating LAIs therapy was poor adherence to oral medication regimens (96.9%), followed by patient or family requests (74.3%). For monitoring adverse effects, the most common strategies were scheduled follow-up with symptom assessment (94.1%), routine laboratory testing (89.6%), treatment adjustment based on reported adverse effects (89.6%), and comprehensive patient-family communication (84.2%).

The majority of psychiatrists recognized the therapeutic value of LAIs (80.2%). Similarly, 79.9% acknowledged the importance of LAIs in managing severe mental disorders, and 87.8% were optimistic about future applications. This positive sentiment was reflected in their willingness to promote LAIs in clinical practice.

Cost reduction was identified as the most critical priority (88.3%). Other key factors included improving patient and family awareness (80.6%), increasing injection convenience and comfort (71.6%), and early LAIs initiation (within two years of diagnosis; 71.6%). Patient-related stigma was a concern for 64.4% of the psychiatrists. Effective physician-patient communication was considered crucial for successful LAIs promotion by 84.7% of the respondents.

### 3.3. Subgroup analysis: Mann-Whitney U test results

Clinical experience with LAIs emerged as the most influential factor. Compared with those with limited experience, psychiatrists with substantial LAIs experience held significantly more positive attitudes across all dimensions compared to those with limited experience, including perceived therapeutic value (U = 4621, *P* < 0.001, r = 0.165), willingness to promote (U = 4741.5, *P* = 0.047, r = 0.133), and support for early application (U = 4088, *P* = 0.001, r = 0.233). They also demonstrated greater awareness of stigma-related issues (U = 4439, *P* = 0.010, r = 0.173) and appreciation of physician-patient communication (U = 4427, *P* = 0.007, r = 0.182). Furthermore, psychiatrists with >10 years of clinical practice (U = 4869, *P* = 0.003, r = 0.199) and those holding senior professional titles (U = 4323.5, *P* = 0.049, r = 0.132) attributed significantly greater importance to communication for promoting LAIs. Additionally, psychiatrists with >10 years of clinical experience reported stronger support for early LAIs application (U = 4960, *P* = 0.007, r = 0.181). The significant results are detailed in Table 2.

**Table 2:** Comparison of attitudes toward LAIs by psychiatrist characteristics. (Mann-Whitney *U* Test, *n* = 222).

Attitude variable	Grouping variable	*Mann-Whitney U*	Z value	*P-*value*	Effect size (*r*)
Overall value of LAIs in psychiatric treatment	Experienced *vs.* Limited	4621	−2.461	0.014	0.165
Willingness to promote	Experienced *vs.* Limited	4741.5	−1.986	0.047	0.133
Attitude toward early application	Experienced *vs.* Limited	4088	−3.469	0.001	0.233
Encountered stigma issues	Experienced *vs.* Limited	4439	−2.576	0.010	0.173
Role of physician-patient communication	Experienced *vs.* Limited	4427	−2.719	0.007	0.182
Attitude toward early application	>10 *vs*. ≤ 10 years practice	4960	−2.698	0.007	0.181
Role of physician-patient communication	>10*vs.* ≤ 10 years practice	4869	−2.963	0.003	0.199
Role of physician-patient communication	Senior *vs.* Junior title	4323.5	−1.970	0.049	0.132

*: Only results with *P* < 0.05 are shown.

### 3.4. Spearman correlation analysis

Spearman correlation analysis revealed significant positive associations between psychiatrists’ attitudes toward LAIs and both clinical experience and self-assessed knowledge (Table 3). After Benjamini-Hochberg FDR correction, the strongest correlations remained statistically significant: LAIs experience with support for early LAIs use (ρ = 0.302, *q* < 0.01), followed by knowledge level with willingness to promote LAIs (ρ = 0.297, *q* < 0.01). The correlation coefficients between knowledge level and various attitude-related variables ranged from 0.22 to 0.30, and most of these associations remained significant after FDR correction (*q* < 0.05). In contrast, some weaker correlations were no longer significant after FDR adjustment. For example, the association between professional title and perceived overall value of LAIs (ρ = 0.133) did not survive correction (*q* > 0.05). The perceived importance of physician-patient communication remained significantly correlated with all four psychiatrist characteristics examined (all *q* < 0.05). These results indicate that the associations with LAIs prescribing experience and knowledge level were robust to multiple comparison correction, whereas some weaker associations should be interpreted with caution.

**Table 3:** Correlations between psychiatrist characteristics and attitudes toward LAIs (n= 222).

Characteristic	Overall Value	Value in SMI^#^	Future Prospects	Willingness to Promote	Early Use	Stigma Awareness	Communication Importance
Years in practice	0.078	0.154*	0.052	0.063	0.160*	−0.072	0.197**
Professional title	0.133	0.190*	0.017	0.030	0.104	−0.050	0.191**
Knowledge level	0.250**	0.283**	0.173*	0.297**	0.294**	−0.172*	0.280**
LAIs experience	0.217**	0.279**	0.133	0.204**	0.302**	−0.221**	0.253**

#: SMI = Severe Mental Illness; *P* values were adjusted using the Benjamini-Hochberg false discovery rate (FDR) procedure; *q < 0.05, **q < 0.01.

## 4. Discussion

This questionnaire-based study systematically evaluated psychiatrists’ knowledge, attitudes, and willingness to promote LAIs in Fujian Province, China. The findings revealed that the majority demonstrated high awareness of the clinical benefits of LAIs, particularly in terms of enhancing treatment adherence and convenience (94.6%). Additionally, 80.2% recognized the therapeutic value of LAIs, and 87.8% viewed their application prospects positively. Furthermore, 82.4% expressed a willingness to promote LAIs. However, high cost was consistently identified as the primary barrier to wider adoption (88.3%). Subgroup and correlation analyses further revealed that direct clinical experience with LAIs was the most influential factor fostering positive attitudes and willingness to promote them, with stronger associations than with knowledge alone.

The generally favorable perception of LAIs observed aligns with recent international research, although regional and policy-related differences exist. For instance, a European survey indicated that most mental health practitioners held positive views toward LAIs, with approximately 40% supporting use in first-episode patients [28]. Similarly, a survey by Ciglar et al. reported that 93.6% of psychiatrists recognized the significant role of LAIs in schizophrenia treatment, and 81.3% favored treatment regimens based on LAIs [29]. Additionally, a recent survey by Jin et al. among community psychiatrists in Beijing revealed that more than 80% would select LAIs for patients with poor adherence, limited home monitoring, or an elevated risk of adverse incidents [25].

In this study, more than 70% of the physicians supported early LAIs initiation (within two years of diagnosis), which is higher than the rate reported in earlier international surveys [30,31]. This increased receptivity may be attributed to recent national policy in China, including the dissemination of Guidelines for the Management and Treatment of Severe Mental Disorders and the implementation policies for free LAI medication in several regions [12,32]. These initiatives have likely increased clinician exposure to and familiarity with LAIs, fostering more positive attitudes.

Nevertheless, previous studies have documented more cautious or even negative perspectives in other settings, highlighting the impact of contextual factors. For instance, an earlier Asia-Pacific survey revealed that only approximately one-third of psychiatrists considered switching to LAIs as the preferred strategy for nonadherence, with significant regional variation: 25% in Hong Kong, 30% in Australia, and 47% in Malaysia [33]. In contrast, research by Oguchi et al. in Japan showed that over half of psychiatrists held unfavorable views toward LAIs [23]. These varying attitudes likely reflect differences in national healthcare systems, medication reimbursement policies, SGA-LAIs availability and market duration across regions, potential overestimation of oral antipsychotic adherence [34] and variations in study methodologies and participant demographics.

This study identified direct clinical experience with LAIs as a key factor shaping positive attitudes, suggesting a target for intervention. We observed that physicians with direct usage experience demonstrated significantly higher scores across all positive dimensions, including willingness to promote and perceived value (*P* < 0.05). Furthermore, correlation analysis indicated that experience exhibited the strongest association with support for early application (ρ = 0.302, *q* < 0.01). These findings suggest that accumulated clinical exposure can effectively mitigate concerns regarding efficacy and safety while enhancing prescribing confidence. This aligns with findings from a recent European survey, which found that experienced LAIs prescribers reported no increased stress when LAIs were recommended, corresponding to a 59.6% increase in LAIs prescriptions over five years [35]. The present survey also assessed clinicians’ ratings of LAI-related adverse effects, including metabolic syndrome, extrapyramidal symptoms, and tardive dyskinesia. Clinicians expressed concern that if patients could not tolerate these effects, the relatively high residual drug exposure and the inability to discontinue treatment immediately might result in patient harm. In line with these concerns, other studies have documented negative attitudes arising from apprehensions about adverse effects, the irreversibility of injection-based treatment, and knowledge deficits [36]. A qualitative study involving focus group discussions with 24 psychiatrists across four Canadian provinces concluded that psychiatrists had limited understanding of LAIs adverse effects, efficacy, and potential outcomes, contributing to greater reservations and reduced prescription [37]. With expanding evidence for SGA-LAIs [38,39] and support from multidisciplinary teams including clinical pharmacists [40], these knowledge barriers can be addressed through structured training and clinical guidance. Consequently, establishing safe, positive initial prescribing experiences and empowering clinicians, particularly early-career psychiatrists, through mentorship, teaching clinics, and case-sharing initiatives may be essential for overcoming clinical inertia and translating awareness into practice.

Economic factors represent the most prominent systemic barrier, underscoring the decisive influence of payment policies on medical technology diffusion and highlighting the importance of adjusting health insurance policies and conducting pharmacoeconomic evaluations. Specifically, 90.5% of the respondents cited high cost as a major clinical barrier, and 88.3% identified price reduction as the primary requirement for broader promotion. These results are consistent with studies from various studies [21,41]. A 10-year mirror-image study increased evidence supporting the use of paliperidone palmitate in the long-term treatment of patients with schizophrenia or schizoaffective disorder to achieve cost savings [42]. A systematic review and meta-analysis indicated that LAIs may improve medication adherence and reduce rates of hospitalization and emergency department visits [43]. Lower medical costs offset higher pharmacy costs, resulting in no statistically significant difference in total healthcare costs. Taken together, these findings provide strong evidence for the real-world comparison of the clinical and economic benefits of LAIs versus oral antipsychotics in the treatment of schizophrenia. Although SGA-LAIs had demonstrated clear pharmacoeconomic advantages in the treatment of mental disorders [44,45], high upfront costs remain a practical obstacle to physician prescribing and patient acceptance [46,47]. A recent multinational survey of 466 psychiatrists revealed that cost and fear of injections were the two most significant barriers to promotion, with notable regional variation [21]. The percentage of Chinese physicians viewing cost as a major barrier (72.0%) far exceeded that in the U.S. (47.6%), France (8.4%), and Spain (11.1%). This disparity likely reflects differences in health insurance reimbursement rates, out-of-pocket payment capacity, and regional economic level. Instead, targeted adjustments to health insurance policies, such as increasing reimbursement rates and refining payment methods, along with establishing multichannel subsidy mechanisms and enhancing pharmacoeconomic evidence, are essential prerequisites [17].

Notably, although clinical experience, knowledge, and attitudes were significantly correlated, their effect sizes were moderate. This implies that among psychiatrists with relatively high baseline knowledge, the potential for further substantial improvement in attitudes may be limited. Furthermore, this finding reinforces that prescribing behavior results from a complex interplay of factors beyond clinical judgment, including patient preferences, insurance structures, and institutional protocols [48,49]. This emphasizes the need for multifaceted intervention strategies.

The study also highlights the important role of communication skills. A large majority of participants (84.7%) viewed effective physician-patient communication as crucial for LAIs promotion, and its perceived importance correlated positively with years of practice, professional title, and clinical experience. Enhancing training in patient-centered communication and fostering shared decision-making can improve patients’ perceptions and acceptance of injectable therapies, reduce treatment-related stigma, and ultimately improve treatment adherence [50,51].

This study has several limitations that should be considered when interpreting the results. First, the cross-sectional design precludes the establishment of causal relationships. For example, although a significant association was observed between clinical experience with LAIs and positive attitudes, the direction of this relationship is unclear. It is uncertain whether accumulated experience fosters favorable attitudes or whether preexisting positive attitudes motivate psychiatrists to seek greater experience with LAIs. Furthermore, unmeasured confounders, such as institutional culture, the availability of continuing medical education, or exposure to pharmaceutical marketing, could simultaneously influence both experience levels and attitudinal outcomes. Second, the use of convenience sampling introduces potential selection bias. Participants recruited from a provincial psychiatry conference may have had greater interest in or familiarity with LAIs than the broader psychiatric community did, particularly psychiatrists in primary care or rural settings and under resourced facilities [52,53], limiting generalizability. Third, the survey instrument relied primarily on closed-ended items, which may not capture nuanced variations in attitude strength or precisely rank the relative importance of specific barriers (e.g., whether high cost is an absolute deterrent or a concern weighed against other factors). Moreover, the questionnaire items were reviewed and adjusted by the research team together with an expert panel, who considered the items relevant. However, the limited size of the expert panel may have reduced the comprehensiveness of content validation. Fourth, the study is susceptible to common method bias, as all data were self-reported at one time point, which may have been influenced by social desirability bias. Although anonymity and clear phrasing were ensured, this approach may inflate observed correlations. Fifth, the operational definition of early use (within two years of diagnosis) included patients who may have already started oral therapy. We did not separately analyze LAIs initiation at first diagnosis; therefore, comparisons with studies focusing exclusively on first-episode psychosis patients should be made cautiously. Finally, although we applied FDR correction to control for multiple comparisons, the causal direction of these correlations cannot be determined, and some weaker correlations were no longer significant after correction and should be interpreted with caution.

Future research should employ longitudinal designs to track changes in psychiatrists’ attitudes following policy reforms or initial LAI prescription experiences, and validate the current findings in larger, more representative samples across diverse practice settings. A dedicated scale for measuring attitudes toward LAIs should be developed and validated using confirmatory factor analysis and known-groups comparisons, ideally through mixed methods (e.g., Likert-scale items combined with qualitative interviews) to better quantify attitude intensity and explore underlying drivers. More granular subgroup analyses are needed to clarify pivotal clinical concepts such as early-phase application. To improve content validity, future studies should include a broader and more diverse expert panel, including community practitioners and clinical pharmacists. Stratified random sampling across different hospital levels and geographic regions will help obtain more representative data. Finally, a priori sample size calculations based on the effect sizes observed in this study should be performed to ensure adequate statistical power, particularly for subgroup analyses with smaller expected effect magnitudes.

## 5. Conclusion

The results of this cross-sectional study demonstrate that psychiatrists in Fujian Province, China, generally recognize the clinical benefits of LAIs, particularly their role in enhancing medication adherence and patient convenience. They also expressed favorable attitudes toward the overall therapeutic value and future application potential of LAIs, reflected in strong willingness to promote their use. However, two primary barriers limit broader LAIs adoption. First, personal clinical experience is the most influential factor associated with both positive attitudes and willingness to promote LAIs. Second, economic factors were consistently cited as the primary systemic barrier. Comprehensive strategies, such as creating positive initial prescribing experiences for psychiatrists, reducing costs, and conducting real-world pharmacoeconomic evaluations, are essential to bridge the gap between awareness and practice, thereby improving treatment outcomes and quality of life for patients with mental health disorders.

## Supporting information

S1 File Survey on LAI.docx. The full survey questionnaire used in this study.
(DOCX)

## Abbreviations

LAI: Long-acting injectable
SGA-LAI: Second-generation antipsychotic long-acting injectable
