# Supplementary material for: Perceptions of psychiatrists toward long-acting injectable antipsychotics: A cross-sectional survey from Fujian, China
Source: PLoS One. 2026 Nov 2;21(11):e0362055. doi: 10.1371/journal.pone.0362055 (PMC13726915; doi:10.1371/journal.pone.0362055)
Supplement: Supplementary file 1 [file pone.0362055.s001.docx]

**Survey on Psychiatrists' Willingness and Attitudes Toward the Use of Long-Acting Injectable Antipsychotics (LAIs)**

1.Gender: [Single choice]

| **Option** | **Count** | **Percentage** |
| --- | --- | --- |
| Male | 132 | 59.46% |
| Female | 90 | 40.54% |
| Valid responses | 222 |  |

2. Age [Single choice]

| **Option** | **Count** | **Percentage** |
| --- | --- | --- |
| A: ≤29 years | 34 | 15.32% |
| B: 30–39 years | 104 | 46.85% |
| C: 40–49 years | 52 | 23.42% |
| D: 50–59 years | 30 | 13.51% |
| E: ≥60 years | 2 | 0.90% |
| Valid responses | 222 |  |

3.Years of clinical experience [Single choice]

| **Option** | **Count** | **Percentage** |
| --- | --- | --- |
| 5 years or less | 51 | 22.97% |
| 6–10 years | 57 | 25.68% |
| 11–20 years | 62 | 27.93% |
| More than 20 years | 52 | 23.42% |
| Valid responses | 222 |  |

4.Professional title [Single choice]

| **Option** | **Count** | **Percentage** |
| --- | --- | --- |
| A. Chief physician | 12 | 5.41% |
| B. Associate chief physician | 53 | 23.87% |
| C. Attending physician | 86 | 38.74% |
| D. Resident physician | 71 | 31.98% |
| Valid responses | 222 |  |

5.How familiar are you with long-acting injectable antipsychotics (LAIs)? [Single choice]

| **Option** | **Count** | **Percentage** |
| --- | --- | --- |
| A. Very familiar | 70 | 31.53% |
| B. Somewhat familiar | 144 | 64.86% |
| C. Heard of them but not very familiar | 7 | 3.15% |
| D. Not at all familiar | 1 | 0.45% |
| Valid responses | 222 |  |

6.Compared with oral medications, what advantages do LAIs have in treating psychiatric disorders? (Select all that apply) [Multiple choice]

| **Option** | **Count** | **Percentage** |
| --- | --- | --- |
| A. Better medication adherence | 207 | 93.24% |
| B. More stable plasma drug levels | 147 | 66.22% |
| C. Reduced dosing frequency, more convenient for patients | 210 | 94.59% |
| D. Fewer side effects | 68 | 30.63% |
| Valid responses | 222 |  |

7.What limitations or problems do you perceive with LAIs in clinical practice? (Select all that apply) [Multiple choice]

| **Option** | **Count** | **Percentage** |
| --- | --- | --- |
| A. Injection pain | 84 | 37.84% |
| B. Some patients do not accept the injection route | 134 | 60.36% |
| C. High cost | 201 | 90.54% |
| D. Patients need to visit the hospital/clinic regularly for injections, increasing burden | 96 | 43.24% |
| Valid responses | 222 |  |

8. Have you ever used LAIs in your clinical work? [Single choice]

| **Option** | **Count** | **Percentage** |
| --- | --- | --- |
| A. Often | 27 | 12.16% |
| B. Occasionally | 119 | 53.60% |
| C. Rarely | 55 | 24.77% |
| D. Never | 21 | 9.46% |
| Valid responses | 222 |  |

9.If you have used LAIs, under what circumstances would you consider using them? (Select all that apply) [Multiple choice]

| **Option** | **Count** | **Percentage** |
| --- | --- | --- |
| A. Poor adherence to oral medications | 215 | 96.85% |
| B. Severe illness requiring more stable drug control | 97 | 43.69% |
| C. Contraindications or intolerance to oral medications | 150 | 67.57% |
| D. Patient and family actively request LAIs | 165 | 74.32% |
| Valid responses | 222 |  |

10. How do you monitor and manage potential adverse reactions when using LAIs? (Select all that apply) [Multiple choice]

| **Option** | **Count** | **Percentage** |
| --- | --- | --- |
| A. Regular follow-up, asking about symptoms | 209 | 94.14% |
| B. Ordering necessary laboratory tests | 199 | 89.64% |
| C. Adjusting treatment based on severity of adverse reactions | 199 | 89.64% |
| D. Thorough communication with patients and families, providing coping advice | 187 | 84.23% |
| Valid responses | 222 |  |

11. How would you rate the overall value of LAIs in psychiatric treatment? [Single choice]

| **Option** | **Count** | **Percentage** |
| --- | --- | --- |
| A. Very high | 29 | 13.06% |
| B. High | 149 | 67.12% |
| C. Moderate | 43 | 19.37% |
| D. Low | 1 | 0.45% |
| E. Very low | 0 | 0% |
| Valid responses | 222 |  |

12. How would you rate the overall value of LAIs in the management of severe mental illness? [Single choice]

| **Option** | **Count** | **Percentage** |
| --- | --- | --- |
| A. Very high | 39 | 17.57% |
| B. High | 136 | 61.26% |
| C. Moderate | 47 | 21.17% |
| D. Low | 0 | 0% |
| E. Very low | 0 | 0% |
| Valid responses | 222 |  |

13. What do you think is the future outlook for LAIs in psychiatry? [Single choice]

| **Option** | **Count** | **Percentage** |
| --- | --- | --- |
| A. Very promising | 64 | 28.83% |
| B. Somewhat promising | 131 | 59.01% |
| C. Moderate | 25 | 11.26% |
| D. Somewhat limited | 1 | 0.45% |
| E. Very limited | 1 | 0.45% |
| Valid responses | 222 |  |

14. Would you be willing to try and promote LAIs more in your future clinical practice? [Single choice]

| **Option** | **Count** | **Percentage** |
| --- | --- | --- |
| A. Very willing | 58 | 26.13% |
| B. Somewhat willing | 125 | 56.31% |
| C. Unsure | 35 | 15.77% |
| D. Not very willing | 3 | 1.35% |
| E. Not willing at all | 1 | 0.45% |
| Valid responses | 222 |  |

15. What do you think is the most urgent issue to address in promoting LAIs? (Select all that apply) [Multiple choice]

| **Option** | **Count** | **Percentage** |
| --- | --- | --- |
| A. Improve awareness and acceptance among patients and families | 179 | 80.63% |
| B. Lower the cost of LAIs | 196 | 88.29% |
| C. Strengthen clinical research and evidence base for LAIs | 157 | 70.72% |
| D. Improve injection convenience and comfort | 159 | 71.62% |
| Valid responses | 222 |  |

16. What is your opinion on using LAIs early in the disease course (within 2 years of diagnosis)? [Single choice]

| **Option** | **Count** | **Percentage** |
| --- | --- | --- |
| A. Strongly support | 52 | 23.42% |
| B. Support | 107 | 48.20% |
| C. Neutral | 56 | 25.23% |
| D. Oppose | 7 | 3.15% |
| E. Strongly oppose | 0 | 0% |
| Valid responses | 222 |  |

17. In your clinical practice, have you encountered stigma against patients due to the use of LAIs? [Single choice]

| **Option** | **Count** | **Percentage** |
| --- | --- | --- |
| A. Often | 12 | 5.41% |
| B. Occasionally | 54 | 24.32% |
| C. Rarely | 77 | 34.68% |
| D. Never | 79 | 35.59% |
| Valid responses | 222 |  |

18. How important do you think doctor‑patient communication is in promoting the use of LAIs? [Single choice]

| **Option** | **Count** | **Percentage** |
| --- | --- | --- |
| A. Very important | 74 | 33.33% |
| B. Important | 114 | 51.35% |
| C. Moderately important | 32 | 14.41% |
| D. Slightly important | 2 | 0.90% |
| E. Not important at all | 0 | 0% |
| Valid responses | 222 |  |
